# Prolotherapy agent P2G is associated with upregulation of fibroblast growth factor-2 genetic expression in vitro

**DOI:** 10.1186/s40634-020-00312-z

**Published:** 2020-12-06

**Authors:** Elisha Johnston, Chandrakanth Emani, Andrew Kochan, Kidane Ghebrehawariat, John Tyburski, Michael Johnston, David Rabago

**Affiliations:** 1 Palos Verdes Peninsula High School, 27118 Silver Spur Rd, Rolling Hills Estates, CA 90274 USA; 2 Department of Biology, Western Kentucky University, 1906 College Heights Blvd, Bowling Green, KY 42101-1080 USA; 3 Healing Arts Research, 4835 Van Nuys Blvd # 100, Sherman Oaks, CA 91403 USA; 4 Independent Researcher, Pittsburgh, PA 15208 USA; 5 Nelson Scientific Labs LLC, 44790 Maynard SQ, Ashburn, VA 20147 USA; 6 Independent Researcher, 5727 Ravenspur Dr. #309, Rancho Palos Verdes, CA 90275 USA; 7 Department of Family and Community Medicine, Penn State College of Medicine, Hershey, PA 17033 USA

## Abstract

**Purpose:**

Osteoarthritis (OA) is a prevalent, progressively degenerative disease. Researchers have rigorously documented clinical improvement in participants receiving prolotherapy for OA. The mechanism of action is unknown; therefore, basic science studies are required. One hypothesized mechanism is that prolotherapy stimulates tissue proliferation, including that of cartilage. Accordingly, this in vitro study examines whether the prolotherapy agent phenol-glycerin-glucose (P2G) is associated with upregulation of proliferation-enhancing cytokines, primarily fibroblast growth factor-2 (FGF-2).

**Methods:**

Murine MC3T3-E1 cells were cultured in a nonconfluent state to retain an undifferentiated osteochondroprogenic status. A limitation of MC3T3-E1 cells is that they do not fully reproduce primary human chondrocyte phenotypes; however, they are useful for modeling cartilage regeneration in vitro due to their greater phenotypic stability than primary cells. Two experiments were conducted: one in duplicate and one in triplicate. Treatment consisted of phenol-glycerin-glucose (P2G, final concentration of 1.5%). The results were assessed by quantitative Reverse Transcriptase-Polymerase Chain Reaction (qRT-PCR) to detect mRNA expression of the FGF-2, IGF-1, CCND-1 (Cyclin-D), TGF-β1, AKT, STAT1, and BMP2 genes.

**Results:**

P2G - treated preosteoblasts expressed higher levels of FGF-2 than water controls (hour 24, *p* < 0.001; hour 30, *p* < 0.05; hour 38, *p* < 0.01). Additionally, CCND-1 upregulation was observed (*p* < 0.05), possibly as a cellular response to FGF-2 upregulation.

**Conclusions:**

The prolotherapy agent P2G appears to be associated with upregulation of the cartilage cell proliferation enhancer cytokine FGF-2, suggesting an independent effect of P2G consistent with clinical evidence. Further study investigating the effect of prolotherapy agents on cellular proliferation and cartilage regeneration is warranted.

## Background

Osteoarthritis (OA) is a common, impactful and progressively degenerative disease [8, 14, 46] characterized by cartilage erosion that leads to degradation of joint structure and function [9, 22]. Treatment is supportive and spans a range of modalities [3, 13, 19, 21, 32, 45]. The development of therapy that stimulates cartilage regeneration and controls pain is the subject of active research. A growing number of clinicians across several specialties carry out an injection therapy known as prolotherapy, a term coined from “proliferative” and “therapy” [7]. The current protocols, which were developed in the 1950s [29], comprise multiple small-volume injections of therapeutic solution, usually either hypertonic dextrose (D-glucose) or phenol-glucose-glycerin (P2G), at ligament and tendon entheses and in adjacent joint spaces [51]. Early clinical data [50] and recent clinical trials and meta-analysis data [53] support reduced pain and stiffness and improved function in patients undergoing this treatment. However, the mechanism of action is not well understood. Early researchers observed that animal tissue was hypertrophied following prolotherapy [29]. Physician scientists hypothesize a multifactorial mechanism of action [51], with one specific hypothesis positing that prolotherapy slows OA progression by stimulating cartilage regeneration [31]. This hypothesis is supported by a study of 6 OA patients that used pre- and postarthroscopic imaging and histological staining to show clinical evidence suggesting that HD stimulates joints to regrow cartilage [55].

Clinical researchers have called for more basic science studies on prolotherapy, especially regarding potential cellular and molecular mechanisms of action [51, 53]. Freeman et al. [25] established the field of in vitro prolotherapy with a viability assay and found that P2G induces the proliferation of MC3T3-E1 cells. Another research team used flow cytometry to reproduce the finding that P2G induces the proliferation of MC3T3-E1 cells [34]. Consistent with previous in vitro research on prolotherapy, our study utilized the MC3T3-E1 cell line. Established in 1981, this is a murine nontransformed cell line derived from newborn mouse calvaria [17, 39, 44, 49, 54]. In addition to the specific study of prolotherapy [25, 34], the MC3T3-E1 cell line has been used more generally to study skeletal tissue regeneration [5, 38, 42, 58].

The present study expands the newly emerging field of in vitro prolotherapy by being the first to investigate the molecular mechanisms by which P2G activates cell proliferation, as shown in previous research [25, 34]. The primary focus is fibroblast growth factor-2 (FGF-2) because it facilitates cell proliferation [56]. Using an in vitro model, Chien and colleagues showed that murine cells synthesize FGF-2 [11]. Researchers have further shown in rabbits [15, 36], rats [59], and mice [33] that FGF-2 changes a cell’s gene expression profile from a state of low/nonproliferation to one of increased proliferation. As a downstream marker for proliferation, the current study quantifies mRNA expression of the cell cycle gene Cyclin D1 (CCND-1), which promotes transition from G1 to S stage of the cell cycle [1]. Given the existing evidence for FGF-2 as a factor involved in proliferation, we hypothesize that P2G upregulates FGF-2 and subsequently Cyclin D1. For a broader understanding of the possible mechanisms of P2G as a prolotherapy agent, we also investigated additional genes related to proliferation and regeneration (IGF-1, TGF-B1, BMP-2 and STAT-1).

## Methods

### Experiments

To identify the molecular mechanisms of P2G-induced cell proliferation, two experiments were conducted. Genes targeted in the experiments were identified via a systematic MEDLINE search. The list was narrowed to a primary candidate (FGF-2), a downstream indicator (CCND-1), and four exploratory genes based on published literature and expert recommendations on the subject matter (see Table 1). RPL13A, rather than GAPDH and beta-actin, was utilized as the reference gene for normalizing quantitative Reverse Transcriptase-Polymerase Chain Reaction (qRT-PCR) gene expression data. This choice is supported by several criteria, including (1) a potential effect of experimental treatment (P2G) on housekeeping gene mRNA expression levels [40], (2) an algorithmic analysis of RPL13A, GAPDH, and beta-actin sample performance [4], and (3) published literature indicating that RPL13A is one of the best reference genes for cartilage [6]. We conducted a preliminary experiment in duplicate that demonstrated the usefulness of an experimental protocol from an existing in vitro prolotherapy study [20] and provided independent results. Our primary experiment, conducted in triplicate, utilized a similar approach. The hour 0 measurement of mRNA expression served as a baseline control. Cells were treated for hour 24 with either P2G or cell culture grade water. Cellular mRNA expression was measured at the hour 24 treatment conclusion and then again at hours 30 and 38. mRNA expression of water-treated control cells was also measured in triplicate at hours 0, 24, 30, and 38. The two experiments provided very similar results, and this manuscript only reports the results from the primary water-controlled experiment.

**Table 1:** Primers Selected for PCR Analysis (from PrimerBank)

Gene	Relevance to cartilage	Primer Sequence
FGF-2	Growth factor regulating chondrogenesis and proliferation [12].	Forward: TTAAACGAGTCTTCAAGGTGGTG
		Reverse: GTCCCCAAAGCTCAGGTACTG
CCND-1	Directly promotes the G1/S transition of the cell cycle [66]	Forward: GCGTACCCTGACACCAATCTC
		Reverse: CTCCTCTTCGCACTTCTGCTC
IGF-1	Growth factor regulating proliferation, bone mineralization, and cartilage ECM production [30, 35, 41]	Forward: AGAGGCTACCCGCCTAGTTC
		Reverse: GTACGGAGTAAACACCTGCTC
TGF-β1	Growth factor regulating proliferation and bone formation [35, 59]	Forward: CTGGACTCATCGCAAACACAA
		Reverse: AGGAAGCCTTTGACTTCTGTCTA
BMP-2	Osteoblast differentiation and mineralization [35, 57]	Forward: GGGACCCGCTGTCTTCTAGT
		Reverse: TCAACTCAAATTCGCTGAGGAC
STAT-1	Transcription factor likely involved in mediating FGFR3 [44]	Forward: TCACAGTGGTTCGAGCTTCAG
		Reverse: GCAAACGAGACATCATAGGCA
RPL13A	Housekeeping control gene [53]	Forward: CCCTCCACCCTATGACAAGA
		Reverse: TTCTCCTCCAGAGTGGCTGT

### Cell line

MC3T3-E1 (ATCC Cat #CRL-2594, Subclone 14), a murine nontransformed cell line, was used to study P2G-induced cell proliferation in vitro. The cells were grown as previously reported [25] in a nonconfluent state to allow them to remain undifferentiated osteochondroprogenitors [49].

### Cell culture

Following Freeman [25], we employed a basic in vitro model of articular cartilage by maintaining cell cultures in Dulbecco’s modified Eagle’s medium (high glucose, L. glutamine, sodium pyruvate), along with 10% fetal bovine serum and 1% penicillin-streptomycin. Under normal growth conditions, the cells were cultured in 44 cm^2^ tissue culture dishes (Nest [via FABBX], Rahway, NJ [Cat #: 704001]). For experiments, the cells were seeded in 24-well plates at a density of 26,000 cells per cm^2^ in each well (Nest [via FABBX], Rahway, NJ [Cat #: 702001]). All cultures were incubated at 37 °C in 5% CO_2_.

### Treatment/control

P2G is a solution composed of 2.5% phenol, 25% glycerin, and 25% dextrose in sterile water (Wellness Pharmacy, Birmingham, AL). For treatment, 15 μL of P2G was added to 985 μL of medium in each treatment well of a 24-well tissue culture plate (1.5% P2G final concentration). For the control, a 15 μL aliquot of cell culture grade sterile water was added to each of the control wells (water controls) such that the control wells contained 985 μL medium and 15 μL cell culture-grade water. Water was selected as a control because P2G is mixed in water. For the hour 0 baseline control, the cell samples were collected from the control/treatment wells immediately before treatment initiation. The P2G treatment and water control were applied, and then to facilitate cell proliferation, the samples were incubated for 24 h (adapted from Freeman et al. [25]). At the conclusion of treatment, the cells were washed with 1x phosphate buffered saline. Subsequently, new medium was added to the tissue culture plate. A set of samples of both the treatment and water control cells was collected at 24 h. The cells were incubated again for an additional 6 and 14 h in standard culture medium to enable collection of samples at 30 and 38 h after treatment initiation.

### Messenger RNA extraction and measurement

To examine mRNA levels, cells were lysed, RNA was extracted (1 μg), cDNA was synthesized, and quantitative PCR was carried out using equal amounts of cDNA per sample to measure the expression levels of genes potentially involved in cartilage anabolism. The Qiagen RNeasy Mini kit was used to isolate the mRNA, and DNA levels were quantified using Applied Biosystems High Capacity cDNA Reverse Transcription Kit for cDNA synthesis and SYBR Green. cDNA was amplified by polymerase chain reaction using specific primers (Table 1), and cDNA levels were quantified using a Roche LightCycler 480 II.

### Statistical analysis

Mean differences were compared utilizing statistical techniques in accord with the distributional characteristics of the data. For FGF-2, Welch’s *t*-tests were employed to compare treatment and control groups at each time point because the data were approximately normally distributed (Kolmogorov-Smirnov test with *p*-value = 0.98) and the equality of variance assumption was not reasonable. For IGF-1, two-way ANOVA was employed because the data were approximately normally distributed (Kolmogorov-Smirnov test with *p*-value = 0.6028) and showed relatively equal variances across groups.

The preliminary experiment suggested that P2G treatment is associated with upregulation of FGF-2 in osteochondroprogenitors as early as hour 24. Accordingly, a directional test was performed in the primary water-controlled experiment at hour 30 to investigate whether P2G-treated osteochondroprogenitors exhibit upregulation of a downstream gene regulating cell proliferation (CCND-1) relative to the control. Welch’s *t*-test was employed for CCND-1 because the data were approximately normally distributed (Kolmogorov-Smirnov test with *p*-value = 0.8531) and the equality of variance assumption was not reasonable. Exploratory analyses of TGF-β1, BMP-2, and STAT-1 using two-tailed Welch’s *t*-tests were also conducted to detect whether treated cells display higher or lower gene expression at any time point.

In all cases, the level of significance, 0.05, refers to two-sided probability except for the prespecified directional test of CCND-1 at hour 30. Study statistics were conducted with RStudio (version 1.2.1335) and the Windows (10, version 1903) platform. RStudio was also used to generate graphics and Adobe Illustrator was applied to layer in legends and demarcations of statistical significance.

## Results

### P2G-induced stress is associated with increased FGF-2 mRNA expression and Cyclin D upregulation

**Fig. 1:**
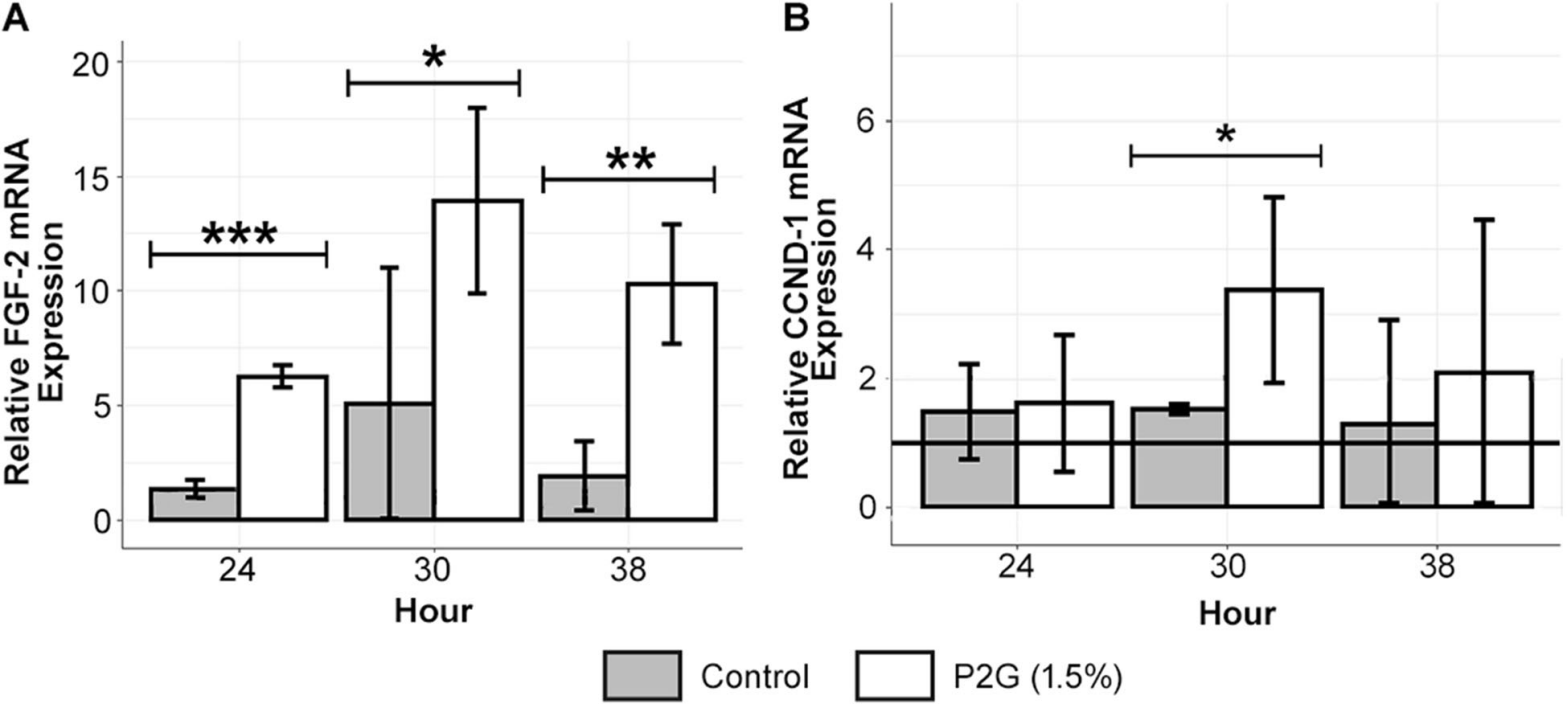
P2G (1.5%) upregulates FGF-2 and CCND-1 mRNA expression in preosteoblasts. **a** Experimental data indicate that relative to water controls, chondrocytes treated with P2G exhibit increased levels of FGF-2 at hours 24, 30, and 38 (Welch’s *t*-tests). At hour 30, numerical Welch’s *t*-test result of a statistically significant difference between means takes precedence over the small visual overlap between treatment and control error bars. **b** P2G (1.5%) upregulates CCND-1 (Cyclin D) mRNA expression at hour 30 in preosteoblasts (fold increase of 2.23, directional Welch’s *t*-test on Experiment 2 data). The solid line is the normalized mean of hour 0 pre-treatment baseline measurements. Control refers to study arms treated with water. The graph displays qRT-PCR mRNA expression. NS *p* > 0.05, * *p* < 0.05, ** *p* < 0.01, *** *p* < 0.001

Figure 1a shows that in Experiment 2, P2G-treated osteochondroprogenitors exhibited higher levels of FGF-2 gene expression relative to the water control at hour 24 with a fold ratio of 4.63 (*p* < 0.001), at hour 30 with a fold ratio of 2.74 (*p* < 0.05), and at hour 38 with a fold ratio of 5.33 (*p* < 0.01). The hour 30 treatment/control 95% confidence interval error bars overlapped, but the difference continued to be statistically significant (*p* < 0.05) [16, 41]. Figure 1b presents evidence that osteochondroprogenitors treated with P2G display upregulation of mRNA expression of CCND-1, also known as Cyclin D (*p* < 0.05). Although P2G-treated osteochondroprogenitors did not exhibit an upregulation of CCND-1 at hour 24, by hour 30, higher levels of CCND-1 relative to the control were detected, with a fold ratio of 2.23 (*p* < 0.05). CCND-1 gene expression returned to normal levels by hour 38.

### P2G-induced stress is associated with changes in IGF-1 mRNA expression

**Fig. 2:**
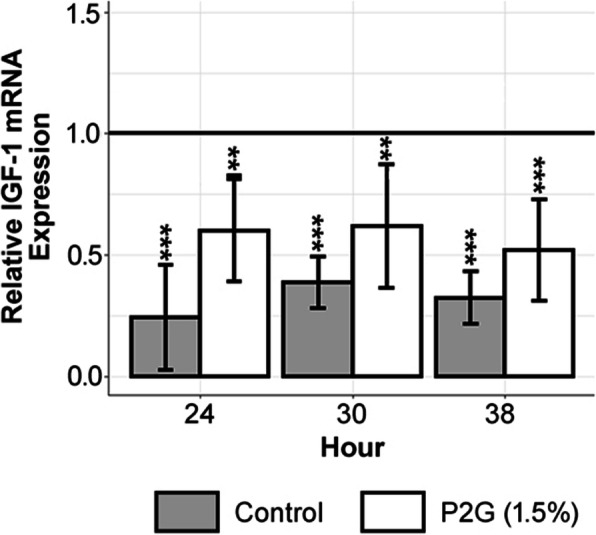
Experimental data indicate that preosteoblasts’ mean relative IGF-1 expression at the pre-treatment baseline is higher than either P2G or water treated preosteoblasts’ mean relative IGF-1 expression at each time point (regression with indicator variables). Experimental data indicate that treated preosteoblasts have a higher mean relative IGF-1 expression than water treated controls (fold increase of 1.82, two-way ANOVA that aggregates the three biological replicates from each time point into an overall study arm and as a consequence precisely estimates the standard error). Significance of each group, as shown with asterisks refers to comparison with the hour 0 control. The solid line is the normalized mean of hour 0 pre-treatment baseline measurements. Control refers to study arms treated with water. The graph displays qRT-PCR mRNA expression. NS *p* > 0.05, * *p* < 0.05, ** *p* < 0.01, *** *p* < 0.001

As illustrated in Fig. 2, P2G-treated osteochondroprogenitors expressed lower mean relative IGF-1 mRNA levels than hour 0 untreated baseline cells (hour 24, *p* < 0.01; hour 30, *p* < 0.01; hour 38, *p* < 0.001). Additionally, Fig. 2 shows diminished IGF-1 expression in water-treated cells across all time points (hour 24, *p* < 0.001; hour 30, p < 0.001; hour 38, *p* < 0.001). Finally, the size of the error bars in Fig. 2 is relatively consistent, which favors pooled testing for a more reliable and precise test. Additionally, two-way ANOVA with interaction terms revealed no significant interaction between hour and treatment (data not shown). In other words, a constant treatment effect over time, starting at hour 24 and persisting through hours 30 and 38, was observed. Accordingly, the more appropriate statistical test is a two-way ANOVA without a main effect for time [hour]. This test indicated a highly significant effect for treatment (1.82-fold increase; *p* < 0.001, not shown). When the preplanned, more fully specified two-way ANOVA with an interaction term for time-specific comparisons between P2G and water control was fit to the data, the model produced estimates that included a 2.47-fold increase of IGF-1 mRNA expression at hour 24 (*p* < 0.01) but nonsignificant increases at hours 30 (1.59-fold increase, *p* = 0.0576) and 38 (1.61-fold increase, *p* = 0.0977).

### P2G-induced stress possibly Upregulates TGF-β1 but not BMP-2 or STAT-1 gene expression

**Fig. 3:**
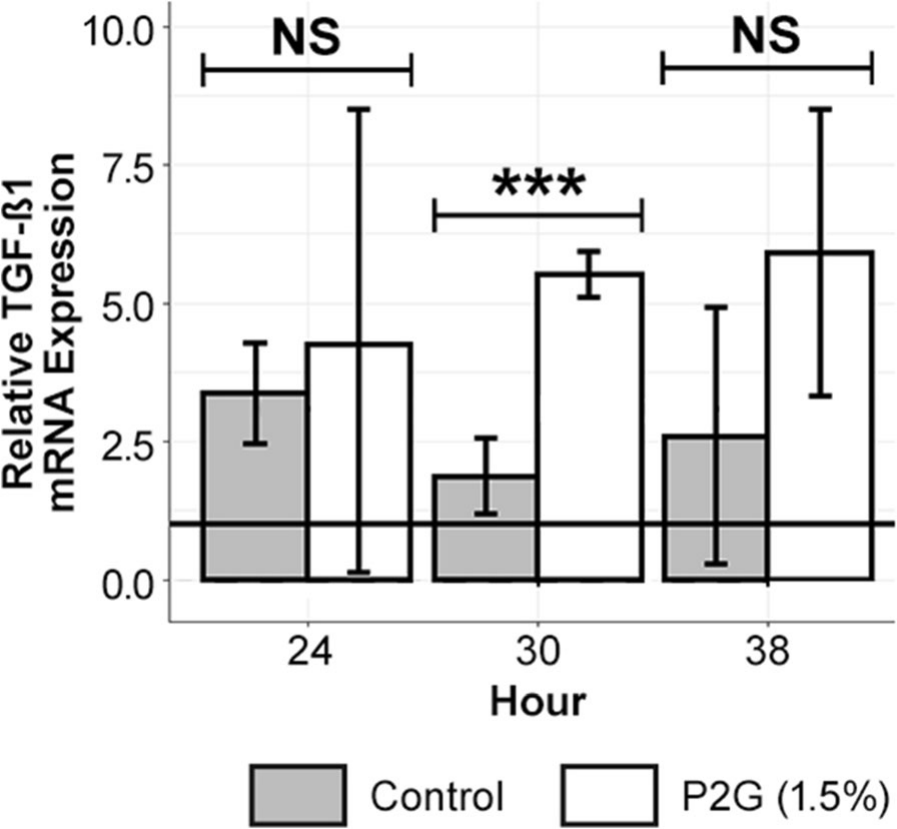
Exploratory investigations suggest P2G possibly effects preosteoblasts’ TGF-β1 mRNA expression. Experimental data, which includes water controls, suggests that P2G may upregulate TGF- β1 gene expression at hour 30 (Welch’s *t*-tests). The solid line is the normalized mean of hour 0 pre-treatment baseline measurements. Control refers to study arms treated with water. The graph displays qRT-PCR mRNA expression. NS *p* > 0.05, * *p* < 0.05, ** *p* < 0.01, *** *p* < 0.001

Figure 3 indicates that at hour 30, P2G-treated osteochondroprogenitors exhibited higher levels of TGF-β1 gene expression relative to the water control, with a fold ratio of 1.26 (*p* < 0.001). In contrast, at hours 24 and 38, P2G-treated osteochondroprogenitors exhibited expression levels of TGF- β1 similar to those in the water control. Moreover, the water-controlled experiment did not yield any evidence of a significant difference in BMP-2 and STAT-1 gene expression between the treatment and control at 24, 30, or 38 h (data not shown).

## Discussion

This study provides evidence that when P2G is applied to MC3T3-E1 cells, the treatment activates FGF-2-specific proliferation-related gene expression, changes neither BMP-2 nor STAT-1 expression, and produces time-dependent activation of IGF-1 and TGF-β1 gene expression patterns.

The finding that P2G upregulates FGF-2 is directly supported by both our preliminary and primary experiments, each of which shows that P2G treatment is followed by increased levels of FGF-2 mRNA expression. Further supporting this finding is the experimental result indicating that CCND-1 mRNA levels are increased in P2G-treated osteochondroprogenitors relative to a water control. CCND-1 expression advances cells through the G1 checkpoint of the cell cycle, accelerating cell proliferation [47]. Researchers have shown that direct FGF-2 application to cells increases CCND-1 expression through the MAPK pathway [23, 24]. Others have shown, both in vitro [56] and in vivo [37], that FGF-2 induces cell proliferation, suggesting that P2G-induced upregulation of FGF-2 mRNA may lead to cell proliferation. The finding that CCND-1 upregulation after FGF-2 upregulation at 24 h aligns with the following previously published research. CCND-1 upregulation suggests that P2G-treated osteochondroprogenitors proliferate between 33 and 45 h after treatment initiation first through FGF-2 and then CCND-1 [34]. The return of CCND-1 levels to normal at hour 38 is consistent with the long-established finding that CCND-1 is highly regulated to prevent uncontrolled cell division. A clinical study showing that prolotherapy stimulates cartilage growth [55] highlights its potential value for future research regarding the role of FGF-2 and CCND-1 in inducing proliferating cells to deposit ECM to heal OA. The prolotherapy agent P2G may induce chondrocytes to upregulate FGF-2, which leads to downstream upregulation of CCND-1, inducing cells to proliferate, a finding previously reported in two independent studies [25, 34]. Overall, these findings suggest that FGF-2 mediated activation of CCND-1 is a biological mechanism by which a prolotherapy agent induces cell proliferation. This basic science finding provides evidence to support preclinical prolotherapy research that explores potential processes by which a prolotherapy agent may induce cell proliferation and cartilage regeneration in models that are physiologically closer to humans [48].

The study results also suggest that P2G induces an early response and time-dependent effect on IGF-1 gene expression. The effect occurs within the context that osteochondroprogenitors, regardless of treatment with P2G or water, exhibit decreased levels of IGF-1 compared to untreated baseline. Understanding this finding of attenuated IGF-1 expression may require a different research design with multiple controls at each time point. At the 24-, 30-, and 38- h time points, P2G-treated cells expressed more FGF-2 and IGF-1 mRNA than water-treated (control) cells (hour 24: *p* < 0.01, hour 30: *p* = 0.0576, hour 38: *p* = 0.0977). Moreover, IGF-1 mRNA expression levels at hour 38 were lower those at hour 24, which is consistent with prior literature showing IGF-1 acts as an immediate early gene in its osteogenic role [43]. Furthermore, Hughes-Fulford and Li [33], who also used the MC3T3-E1 cell line, found that direct FGF-2 treatment suppresses IGF-1 mRNA expression. This suggests that P2G-induced FGF-2 upregulation may be responsible for the suppression of IGF-1 mRNA expression at hours 30 and 38. In future studies, knocking down FGF-2 mRNA with RNA interference and assaying changes in IGF-1 may be of value to determine the effect of P2G treatment on IGF-1 expression. The evidence from our current study likely indicates that FGF-2, rather than IGF-1, is the more important contributor to cell proliferation.

The results of this study indicate that P2G may induce a very short period of increased TGF-β1 gene expression in osteochondroprogenitors. Ekwueme and colleagues [20] studied TGF-β1 protein expression, suggesting that P2G negatively regulates TGF-β1 signaling. The difference in findings may be the result of a timing/sampling difference in protocols. This current study does not provide evidence that P2G affects expression of BMP-2, a cytokine known to increase cartilage repair under certain conditions and increase ossification under others [52]. STAT-1, which is known to be involved in the global immune response [28], does not seem to be affected by P2G treatment under the study conditions which are focused on the local environment.

This study has limitations. The most relevant is the use of the murine MC3T3-E1 cell line, which is not a human primary chondrogenic cell line. Nonetheless, MC3T3-E1 cells are used for modeling cartilage regeneration and are considered reliable because of their greater phenotypic stability compared to primary cells [17] and retention of an osteochondroprogenitor phenotype in culture [30, 54]. The use of the MC3T3-E1 cell line to study the direct effect of P2G on the expression of proliferation-related genes aligns current results to earlier in vitro prolotherapy studies that utilized MC3T3-E1 cells to directly study proliferation [25, 34]. Examples of recently published articles using the MC3T3-E1 cell line for research on cartilage include those by Li et al. [44], Kang et al. [35], and Cai et al. [10]. As an osteochondroprogenitor, MC3T3-E1 cells represent an earlier developmental stage than chondrocytes, the unique cellular component of cartilage [2, 26]. As chondrocytes are more fully differentiated, the environment may not be as influential in inducing chondrocytes to proliferate; therefore, additional experiments are required to definitively confirm that P2G upregulates FGF-2 in chondrocytes. Prolotherapy studies in vitro also do not entirely reproduce the entire joint environment in a tissue culture dish. For example, MC3T3-E1 cells do not involve any inflammatory stimuli. For this reason and others, in vitro prolotherapy studies will not be able to fully reproduce the in situ environment of an osteoarthritic joint [18, 27, 57]. Regardless, in vitro studies play a vital role in demonstrating cell-type-specific responses. Indeed, the current study on murine cells is an important precursor to mechanistic research with complementary transgenic, knockin, and knockout murine models, preferably humanized, which can help to elucidate the mechanisms by which prolotherapy agents affect gene expression in a complex immune-mediated cellular environment [12].

## Conclusions

The standard of care for OA is supportive and focuses on symptomatic relief [18, 27, 57] rather than slowing or reversing cartilage degradation. This study found that P2G is associated with upregulation of FGF-2 mRNA in osteochondroprogenitors. This is consistent with clinical studies suggesting that prolotherapy stimulates the regeneration of cartilage [55]. Further analyses investigating the effect of prolotherapy agents on cellular proliferation and cartilage regeneration in different cell types and model systems are warranted.
