# Correction to: Prolotherapy agent P2G is associated with upregulation of fibroblast growth factor-2 genetic expression in vitro

**DOI:** 10.1186/s40634-020-00323-w

**Published:** 2020-12-24

**Authors:** Elisha Johnston, Chandrakanth Emani, Andrew Kochan, Kidane Ghebrehawariat, John Tyburski, Michael Johnston, David Rabago

**Affiliations:** 1 Palos Verdes Peninsula High School, 27118 Silver Spur Rd, Rolling Hills, Estates, CA 90274 USA; 2 Department of Biology, Western Kentucky University, 1906 College Heights Blvd, Bowling Green, KY 42101-1080 USA; 3 Healing Arts Research, 4835 Van Nuys Blvd # 100, Sherman Oaks, CA 91403 USA; 4 Independent Researcher, Pittsburgh, PA 15208 USA; 5 Nelson Scientific Labs LLC, 44790 Maynard SQ, Ashburn, VA 20147 USA; 6 Independent Researcher, 5727 Ravenspur Dr. #309, Rancho Palos Verdes, CA 90275 USA; 7 Department of Family and Community Medicine, Penn State College of Medicine, Hershey, PA 17033 USA

**Correction to: J Exp Ortop 7, 97 (2020)**

10.1186/s40634-020-00312-z

Following publication of the original article [1], the below Abstract was missing.

**Abstract**

**Purpose:** Osteoarthritis (OA) is a prevalent, progressively degenerative disease. Researchers have rigorously documented clinical improvement in participants receiving prolotherapy for OA. The mechanism of action is unknown; therefore, basic science studies are required. One hypothesized mechanism is that prolotherapy stimulates tissue proliferation, including that of cartilage. Accordingly, this in vitro study examines whether the prolotherapy agent phenol-glycerin-glucose (P2G) is associated with upregulation of proliferation-enhancing cytokines, primarily fibroblast growth factor-2 (FGF-2).

**Methods:** Murine MC3T3-E1 cells were cultured in a nonconfluent state to retain an undifferentiated osteochondroprogenic status. A limitation of MC3T3-E1 cells is that they do not fully reproduce primary human chondrocyte phenotypes; however, they are useful for modeling cartilage regeneration in vitro due to their greater phenotypic stability than primary cells. Two experiments were conducted: one in duplicate and one in triplicate. Treatment consisted of phenol-glycerin-glucose (P2G, final concentration of 1.5%). The results were assessed by quantitative Reverse Transcriptase-Polymerase Chain Reaction (qRT-PCR) to detect mRNA expression of the FGF-2, IGF-1, CCND-1 (Cyclin-D), TGF-β1, AKT, STAT1, and BMP2 genes.

**Results:** P2G - treated preosteoblasts expressed higher levels of FGF-2 than water controls (hour 24, *p*<0.001; hour 30, *p*<0.05; hour 38, *p*<0.01). Additionally, CCND-1 upregulation was observed (p<0.05), possibly as a cellular response to FGF-2 upregulation.

**Conclusions:** The prolotherapy agent P2G appears to be associated with upregulation of the cartilage cell proliferation enhancer cytokine FGF-2, suggesting an independent effect of P2G consistent with clinical evidence. Further study investigating the effect of prolotherapy agents on cellular proliferation and cartilage regeneration is warranted.

The original article [1] has been corrected.
